# Circulating exosomal miRNA in acute coronary syndrome: mechanisms, clinical relevance and future directions

**DOI:** 10.3389/fmolb.2026.1869889

**Published:** 2026-07-28

**Authors:** P. V. Varshini, Debarati Halder, Tridip Mitra, A. H. Sruthi Anil Kumar, Piyush Agrawal, T. R. Muralidharan, Rajarajeswaran Krishnan, C. M. Dhileeban, S. N. Meenakshi Sundari, Rajiv Janardhanan

**Affiliations:** 1 Division of Medical Research, Faculty of Medicine and Health Sciences, SRM Institute of Science and Technology, Kattankulathur, Tamil Nadu, India; 2 Department of Cardiology, Faculty of Medicine and Health Sciences, SRM Institute of Science and Technology, Kattankulathur, Tamil Nadu, India; 3 Department of Emergency Medicine, Faculty of Medicine and Health Sciences, SRM Institute of Science and Technology, Kattankulathur, Tamil Nadu, India; 4 Department of General Medicine, Faculty of Medicine and Health Sciences, SRM Institute of Science and Technology, Kattankulathur, Tamil Nadu, India

**Keywords:** acute coronary syndrome, clinical prognosis, exosomal miRNAs, myocardial infarction, non-ST-elevation myocardial infarction, ST-elevation myocardial infarction, unstable angina

## Abstract

Despite significant advances in diagnosis and treatment, Acute Coronary Syndrome (ACS) remains one of the leading causes of morbidity and mortality globally. It comprises ST-elevation myocardial infarction, non-ST-elevation myocardial infarction, and unstable angina, caused by complex mechanisms such as plaque rupture, thrombosis, erosion, and microvascular dysfunction. The development of high-sensitivity cardiac troponins has greatly improved the early detection of ACS. However, concerns remain about specificity, timing, and prognostic accuracy. Exosomal miRNAs (ExomiRs) show promise as minimally invasive biomarkers, aiding precise cell communication and protecting encapsulated miRNAs from degradation. During ischemic injury, ExomiRs released from cardiomyocytes act early, often before troponin elevation, and are involved in processes such as inflammation, apoptosis, angiogenesis, fibrosis, and ventricular remodelling. Specific ExomiR signatures have different roles in prognosis, subtype discrimination, and early ACS diagnosis. Future therapeutic potential may include advancements in translational strategies, such as antagomir-based miRNA modulation. Furthermore, the clinical adoption of these biomarkers, despite challenges such as pre-analytical variability, differences across study cohorts, a lack of standardised isolation protocols, and the need for large-scale validation, will significantly improve the sensitivity, specificity, and accuracy of predicting ACS.

## Graphical Abstract

**Figure:**
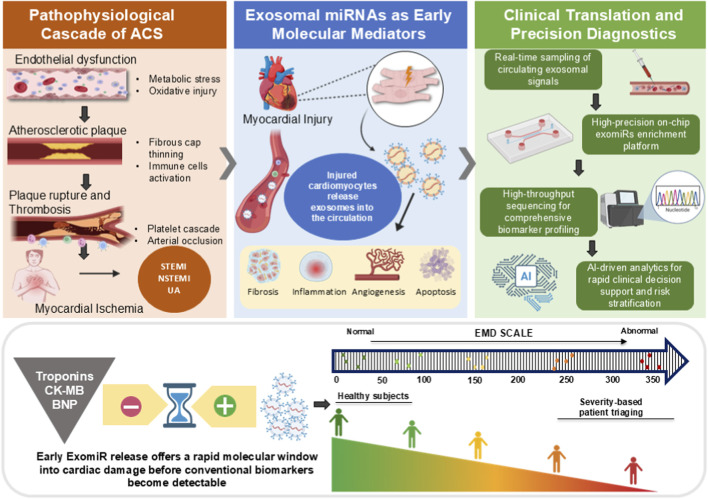
Conceptual overview of exosome-derived microRNAs in acute coronary syndrome, illustrating early molecular release, diagnostic profiling for improved risk stratification and patient management.

## 1 Introduction

Acute Coronary Syndrome (ACS) is a clinical condition characterised by the blockage of a coronary artery, which restricts blood flow to the cardiac muscle (Chen et al., 2013). It comprises a spectrum of acute ischemic disease, including unstable angina (UA) and acute myocardial infarction (AMI), leading to sudden cardiac death (SCD) (Thygesen and Alpert, 2001). ACS constitutes a significant public health concern, with over 0.5 million cases per year and has a substantial economic impact worldwide (Chen et al., 2013). The Global Burden of Disease Study (2022) estimated approximately 315 million cases of Coronary Artery Disease (CAD) globally, with an age-standardised prevalence of 3,605 per 100,000 population (Stark et al., 2024). Significant regional variation exists, with the highest prevalence of ACS in Central Europe, Eastern Europe, and Central Asia, and the lowest in South Asia (Stark et al., 2024). In India, 60.6% of patients presented with STEMI compared with 3.7% in non-ST-elevation myocardial infarction (NSTEMI) or UA, had correlations with modifiable risk factors such as diabetes (30.4%) and hypertension (37.7%), and with non-modifiable risk factors such as smoking (40.2%) (Xavier et al., 2008). Although chest pain is the most commonly reported clinical manifestation of ACS, evidence shows that approximately 30% of ACS patients do not experience chest pain (Roffi et al., 2016). These diagnostic challenges underscore the need for biomarkers that would allow earlier and more accurate detection of ACS. Conventional protein biomarkers are usually elevated at the stage of increased disease severity, thereby limiting the utility of their use for earlier detection of the clinicopathological condition (Chen et al., 2019). Exosomes are small membrane-bound extracellular vesicles secreted by nearly all cell types and are increasingly recognized as important mediators of intercellular communication (Zhang et al., 2020). These vesicles carry a wide range of bioactive molecules, including proteins, lipids, messenger RNAs, and microRNAs (miRNAs), which reflect the physiological or pathological state of their cells of origin (Zhang et al., 2020). Among these components, exosomal microRNAs (ExomiRs) have gained considerable attention because of their remarkable stability in biological fluids (Valadi et al., 2007) and their crucial role in post-transcriptional gene regulation (Wang et al., 2010). ExomiRs are small non-coding RNA molecules that modulate multiple biological pathways, including inflammation, oxidative stress, endothelial dysfunction, apoptosis, fibrosis, angiogenesis, and immune responses, all of which are critically involved in cardiovascular diseases and ACS (Emanueli et al., 2015).

During the development and progression of ACS, cardiomyocytes, endothelial cells, platelets, macrophages, and vascular smooth muscle cells release exosomes enriched with disease-specific miRNA signatures (Henning, 2021). Alterations in circulating ExomiR profiles often occur before the appearance of overt clinical symptoms or irreversible myocardial injury, making them highly promising candidates for early disease detection (Zhou et al., 2020). Unlike conventional cardiac biomarkers that primarily indicate tissue damage after injury has occurred, ExomiRs may provide insights into the underlying molecular and cellular alterations at earlier stages of disease progression (Reiss et al., 2023). Their high sensitivity, specificity, and accessibility through minimally invasive liquid biopsy approaches further enhance their diagnostic and prognostic potential (Preethi et al., 2022).

Emerging evidence suggests that specific ExomiRs are associated with several cardiac-associated clinicopathological conditions, such as plaque instability (IUBMB Life, 2019), thrombosis (Zhang et al., 2026), myocardial ischemia (Moghiman et al., 2021), and adverse cardiac remodelling (Yu et al., 2025), thereby serving as valuable biomarkers for disease tagging and patient risk stratification in ACS (Emanueli et al., 2015). In addition, ExomiRs may help predict treatment response, recurrence risk, and long-term cardiovascular outcomes (Zheng et al., 2021). Beyond their role as biomarkers, they are also being explored as potential therapeutic targets and bio-delivery tools due to their ability to modulate signalling pathways involved in cardiac injury and repair (Zheng et al., 2021). Therefore, this review aims to consolidate current evidence regarding the biological significance, diagnostic utility, and translational potential of ExomiRs in ACS, while highlighting their future applications in precision cardiovascular medicine.

## 2 Contribution of risk factors and pathophysiological mechanisms underlying acute coronary syndrome

ACS comprises three major clinical entities, ST-elevation myocardial infarction (STEMI), NSTEMI, and UA, which are differentiated based on electrocardiographic (ECG) findings and cardiac biomarker levels (Birnbaum et al., 2014). These conditions arise from the progression, rupture, or erosion of atherosclerotic plaques that impair coronary blood flow (Birnbaum et al., 2014), with the clinical presentation depending largely on the extent of luminal obstruction (Nikus et al., 2010). Non-ST-elevation acute coronary syndrome, which includes NSTEMI and UA, typically results from partial or non-occlusive thrombus formation following plaque disruption (Nikus et al., 2010). NSTEMI is characterised by elevated cardiac biomarkers and ECG abnormalities, whereas UA usually presents with prolonged chest pain at rest or minimal exertion without evidence of myocardial necrosis (Nikus et al., 2010). In contrast, STEMI occurs due to complete coronary artery occlusion caused by a persistent fibrin-rich thrombus and is identified by ST-segment elevation on ECG (Enes Demirel et al., 2019). ACS is influenced by multiple modifiable and non-modifiable risk factors, including smoking (Rosengren et al., 2005; Yagi et al., 2010), tobacco use (Yadav et al., 2010; Esteban et al., 2014), obesity (Bęćkowski et al., 2018), hypertension, dyslipidemia (B et al., 2014), thyrotoxicosis (Anghel et al., 2025), type 2 diabetes mellitus (B et al., 2014), age, male sex (Cheema et al., 2020), and family history (Morgan et al., 2007). Younger individuals are particularly susceptible to lifestyle-related risks such as smoking, cocaine use, and sedentary behaviour (Rosengren et al., 2005; Yagi et al., 2010). Additionally, both overt and subclinical hypothyroidism are associated with increased ACS risk (Arambam et al., 2024; Arambam et al., 2021). Pathophysiologically, STEMI is commonly linked to fibrous cap rupture and lipid core exposure, whereas NSTEMI in younger patients is often associated with plaque erosion and platelet-rich thrombi (Akasaka et al., 2010). Other non-atherosclerotic causes, including myocardial bridging, coronary artery embolism, spontaneous coronary artery dissection, and Takotsubo syndrome, may also contribute to ACS development (Waterbury et al., 2020).

## 3 Exosome biogenesis and miRNA packaging

Exosomes are small, single-membrane vesicles that are approximately 30–200 nm in size (Zhang et al., 2020). They are crucial for intercellular communication and carry a complex molecular cargo of proteins and nucleic acids (Zhang et al., 2020; Rajan et al., 2023). They exhibit cell-specific targeting through cell-surface adhesion molecules, such as integrins and tetraspanins (Zöller, 2009). Generally, exosome biosynthesis initiates in the endosomal system, where the endosomal membrane within multivesicular bodies (MVBs) buds inward to create intraluminal vesicles (ILVs) (Mitra et al., 2023). These MVBs mature and interact with organelles such as mitochondria, the endoplasmic reticulum, the trans-Golgi network, and recycling endosomes (Han et al., 2022). They can either fuse with the plasma membrane to release exosomes (secretory MVBs) or merge with lysosomes for breakdown (degradative MVBs) (Han et al., 2022). The fate of MVBs in promoting exosome secretion is determined by the Rab7 GTPase (Han et al., 2022). After maturation, Ras-related in brain GTPase (Rab GTPases), especially Rab27a/b, and soluble N-ethylmaleimide-sensitive factor attachment protein receptor proteins drive antegrade movement of sMVBs, mediating MVB-plasma membrane fusion (Han et al., 2022). MVBs release ILVs as exosomes when they fuse with the plasma membrane (Hessvik and Llorente, 2018). This process is tightly regulated through both Endosomal Sorting Complex Required for Transport (ESCRT)-dependent and ESCRT-independent pathways (Hessvik and Llorente, 2018). Multiple mechanisms, including the ESCRT pathway, lipid raft-mediated pathway and ceramide-dependent pathway, govern exosomal cargo loading (Hessvik and Llorente, 2018). More importantly, the cargo of exosomes are protected from enzymatic degradation because of the lipid bilayer structure, by ensuring stability during transport (Hessvik and Llorente, 2018).

**FIGURE 1:**
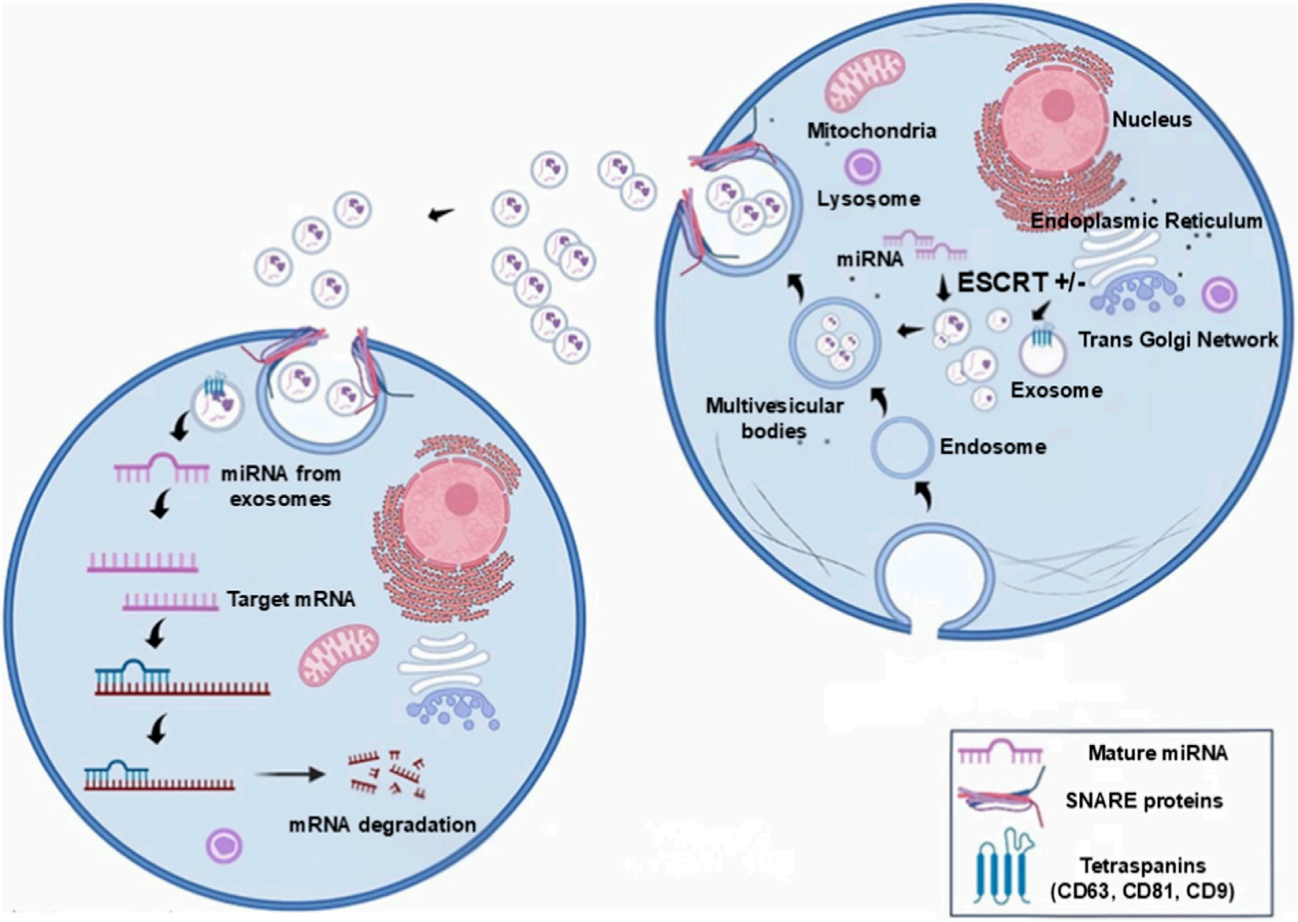
Illustration of the biogenesis, transfer and release of ExomiRs between donor and recipient cells. Exosome formation involves the invagination of Endosomal membrane and development of MVBs. ExomiRs are sorted through ESCRT-dependent or ESCRT-independent pathways and taken up by recipient cells (Immune cells, Endothelial cells), where they bind to target mRNAs and regulate gene expression.

In addition to their structural and functional properties, exosomes transport various miRNAs (Gulati et al., 2024), other small non-coding RNAs such as piwi-interacting RNA, small nuclear RNA, small nucleolar RNA, small Cajal body-specific RNA, and Y RNA, tRNAs and their fragments, mRNAs and their fragments, rRNAs, and long non-coding RNAs (Zeng et al., 2023). The incorporation of miRNAs into exosomes is not random but is regulated (Janas et al., 2015). The ESCRT system along with its components like ESCRT-0, -I, -II, -III, Vacuolar protein sorting-associated protein 4, Vps twenty-associated 1 and Apoptosis-linked gene interacting protein X and ESCRT-independent mechanisms like miRNA-induced silencing complex-mediated, tetraspanins, neutral sphingomyelinase 2, Rab GTPases, are important for MVB development (Janas et al., 2015). Therefore, ubiquitinated cargo proteins (miRNA-protein complexes) are selectively sorted into exosomes by these systems (Janas et al., 2015). Studies reveal that exosomes are highly enriched in specific miRNAs compared with the parent cells (origin cells) (Garcia-Martin et al., 2022). Determination of miRNAs (secreted or retained) will be decided by specific sequences (specific nucleotide motifs-EXOmotifs) like CGGGAG for exosome enrichment, CELLmotifs -AGAAC for intracellular retention and specialized ribonucleoproteins (Garcia-Martin et al., 2022). These pathways can be ribonucleoprotein-dependent or independent (involving lipid rafts) (Wang et al., 2024). Proteins such as Y-box binding protein 1, heterogeneous nuclear ribonucleoprotein A2/B1, synaptotagmin-binding cytoplasmic RNA-interacting protein, fragile X messenger ribonucleoprotein 1, and major vault protein can specifically recognise and bind to miRNA sequences, guiding them into exosomes (Wang et al., 2024) (Figure 1).

## 4 Exosomal miRNAs as early predictors of acute coronary syndrome

Circulating ExomiRs show great potential as early biomarkers and active modulators of cardiac repair in the diagnosis of ACS (Henning, 2021). Cardiac ExomiRs are crucial for intercellular signalling in both homeostasis and disease (Neppl and Wang, 2014). The most frequently evaluated miRNAs, miR-1 and miR-133, act as early and sensitive indicators for the diagnosis of ACS (Kuwabara et al., 2011). A significant elevation of serum ExomiR-146a and miR-122-5p in ACS patients, along with the positive correlations of miR-146a and interleukin-1 beta, interleukin-6 (IL-6), and Tumour Necrosis Factor-α (TNF-α), was identified (Li L. et al., 2021). miR-146a targets the Early Growth Response 1 gene, which plays a role in inflammation, apoptosis, and fibrosis (Ling et al., 2020a). In addition, it exhibits an anti-inflammatory effect by targeting the Tumour Necrosis Factor Receptor-Associated Factor 6 (TRAF6) gene and modulating the IRAK1/TRAF6/NF-κB (Interleukin-1 Receptor-Associated Kinase 1/ Tumour Necrosis Factor Receptor-Associated Factor 6/Nuclear Factor kappa B) pathway (Chen C. et al., 2022). These miRNAs are downregulated in patients with hypertension, obesity, and T2DM, thereby functioning as important predictors for ACS (Roganović, 2021; Ghaffari et al., 2023). Furthermore, miR-4286 levels were significantly associated with adverse lipid profiles, such as low low-density lipoprotein cholesterol (LDL-C) and high triglycerides, thereby elucidating the link between lipid metabolism and disease development (Shen et al., 2021).

Studies reported higher levels of miR-126 and Phosphatase and tensin homolog (PTEN)than miR-21 in serum exosomes from both the UA and AMI groups (Ling et al., 2020b). These miRNAs facilitate smooth muscle proliferation and atherosclerotic plaque formation by suppressing PTEN and activating Protein Kinase B (AKT) signalling (Zhang et al., 2021). Among them, miR-126 inhibits the apoptosis of endothelial cells and promotes angiogenesis by targeting the Phosphatidylinositol 3-Kinase/ Protein Kinase B pathway (Zhang et al., 2021). Its dysregulation in T2DM (Ma et al., 2022) and obesity can be improved by aerobic exercise, thereby enhancing vascular health (Donghui et al., 2019). Plasma miR-208b-3p and miR-143-3p target MYO as a functional target in SCD, identifying them as predictive biomarkers of ACS (Huang et al., 2023). Even though exosomal Nuclear Paraspeckle Assembly Transcript 1 and Matrix metalloproteinase-9 are likely to be upregulated in STEMI patients, no significant alteration of miR-204 expression was observed (Chen et al., 2020).

*In vitro* and *in vivo* experiments proved that comparing traditional biomarkers such as troponins, several ExomiRs, including miR-1, miR-208a, miR-499, and miR-133a, have been reported to be rapidly released into the circulation by downregulating the C-X-C motif chemokine receptor 4 gene (Pinchi et al., 2019; Cheng et al., 2019). These miRNAs get elevated in the plasma of T2DM patients and act as early predictors of diabetic cardiomyopathy (De Gonzalo-Calvo et al., 2017). Moreover, in an assessment of 18 miRNAs identified as diagnostic biomarkers, seven miRNAs (hsa-miR-1227-3p, hsa-miR-5010-5p, hsa-miR-1976, hsa-miR-23b-5p, hsa-miR-1843, hsa-miR-33a-5p and hsa-miR3127-3p) showed a linear relationship with disease progression from CAD to AMI (Guo et al., 2021). Among them, hsa-miR-3615 has been associated with smoking, a modifiable risk factor (Tinè et al., 2023). Based on TargetScan analysis, Secreted Frizzled-Related Protein 1 was identified as a target gene of miR-4516 and miR-203 in AMI patients, thereby suggesting a potential association between elevated expression and disease pathogenesis (Liu et al., 2023). These miRNAs also showed positive correlation with LDL-C, indicating their role as early markers of obesity (Liu et al., 2022). Various risk factors, such as low high-density lipoprotein cholesterol, elevated high-sensitivity C-reactive protein, and elevated systolic blood pressure, were significantly correlated with higher exosomal miR-21-5p (Sahebi et al., 2024), whereas smoking was associated with miR-21-3p levels (Zhu et al., 2019).

**FIGURE 2:**
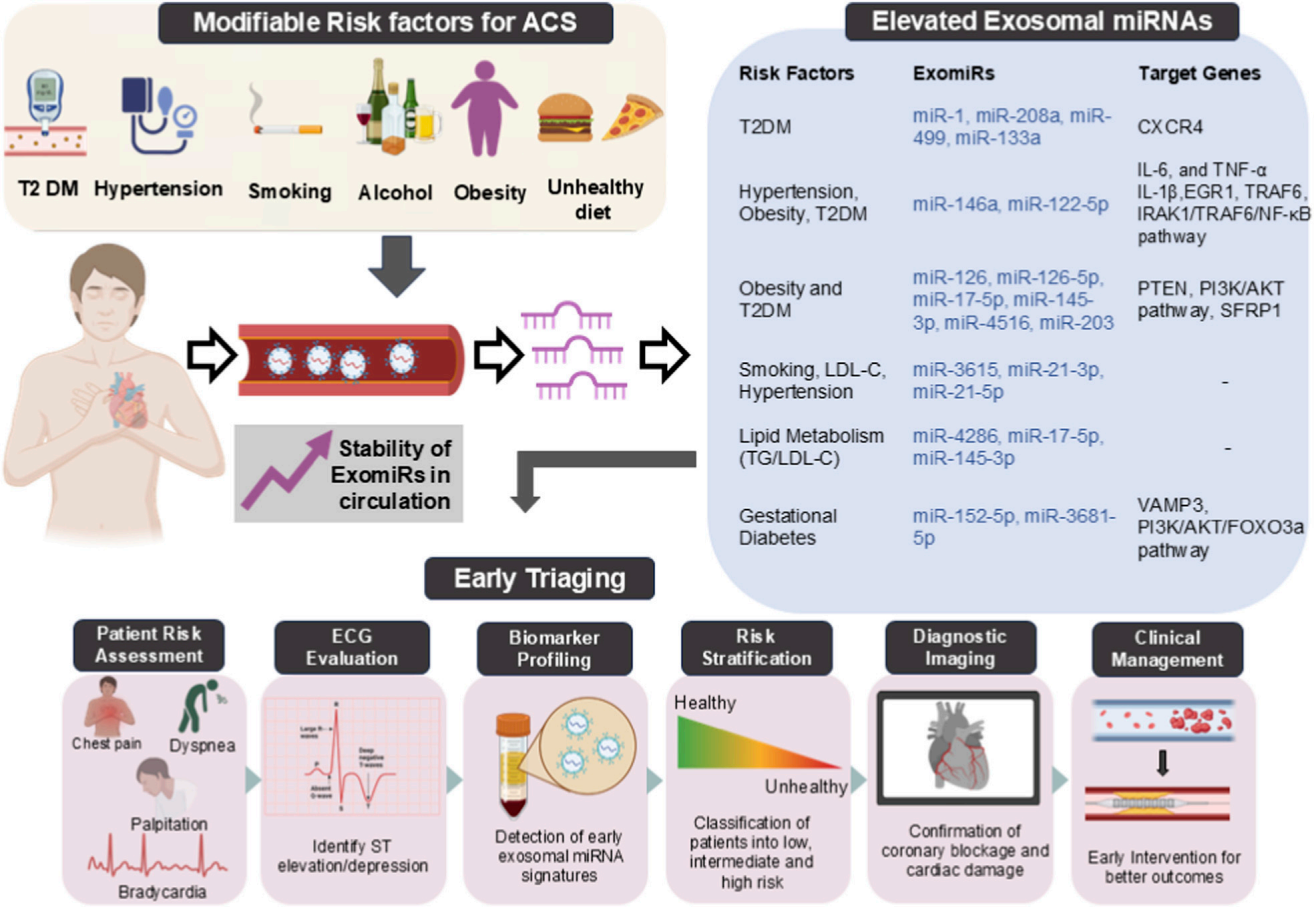
Schematic representation linking ACS risk factors and early triaging workflow. Circulating ExomiRs in ACS are closely associated with modifiable risk factors like diabetes, hypertension, smoking, obesity and unhealthy diet. These ExomiRs regulate key signalling pathways, and their differential expression profiles enable risk stratification in patients with ACS.

Further pieces of evidence demonstrated differences in miR-152-5p and miR-3681-5p expression levels between STEMI and NSTEMI patients (Chen X. et al., 2022). miR-152-5p is upregulated in patients with Gestational Diabetes Mellitus by inhibiting Vesicle-associated membrane protein 3 and the Phosphoinositide 3-kinase/Protein Kinase B/ Forkhead box O3a (PI3K/AKT/FOXO3a) pathway (Zhang et al., 2025). miR-199a-3p and miR-125a-5p exert anti-angiogenic effects during the acute phase of NSTEMI, thereby contributing to disease progression (Joris et al., 2018). Among these, miR-199a-3p plays a role in the Nitric Oxide Synthase/Nitric Oxide pathway in the endothelium by modulating endothelial nitric oxide synthase (eNOS) activity and reducing oxidative stress, and it also acts as an independent risk factor for T2DM (Wang et al., 2018). Additionally, exosomal miR-20b-5p serves as an ideal biomarker of T2DM, which regulates insulin-induced glucose metabolism through AKT signalling (Katayama et al., 2019). Plasma levels of miR-17-5p, miR-126-5p, and miR-145-3p were significantly elevated in ACS patients both before and after percutaneous coronary intervention (PCI) (Xue et al., 2019). They also correlated with clinical parameters, including LDL-C and triglycerides, indicating their significance in obesity and other cardiometabolic conditions (Xue et al., 2019). Circulating miR-20b-5p and miR-1273g-3p may facilitate early diagnosis and prognostic stratification of ACS patients by augmenting existing biomarkers such as cardiac troponin I (cTnI) (Weng et al., 2025). ExomiRs like miR-122, miR-150, miR-16, miR-195, and miR-92a are independently associated with an increased likelihood of ACS, providing complementary predictive value beyond traditional risk factors, including age, gender, smoking, hypertension, obesity, and dyslipidaemia (Elbaz et al., 2020) (Figure 2).

## 5 Stem cell-derived exosomal miRNAs: preclinical insights and therapeutic potential in acute coronary syndrome

ExomiRs derived from stem cells regulate a variety of processes, including oxidative stress, apoptosis, angiogenesis, and fibrosis (Moghaddam et al., 2019). Several preclinical models have exhibited substantial cardioprotective effects of particular miRNAs, like miR-21, miR-22, miR-24, miR-29, miR-126, and miR-2100 (Moghaddam et al., 2019). Mesenchymal stem cells (MSCs) provide cardio-protection during myocardial ischemia/reperfusion injury via exosome secretion (Peng et al., 2020). This effect can be ascribed to the capacity of exosomes to deliver functional miRNAs to damaged cardiac tissue (Peng et al., 2020). For example, miR-25-3p inhibits pro-apoptotic targets such as Fas Ligand and PTEN, and downregulates Enhancer of Zeste Homolog 2 (Peng et al., 2020). This results in activation of the eNOS and Suppressor of Cytokine Signalling 3 pathways, promoting anti-apoptotic and anti-inflammatory responses in ischemic myocardium (Peng et al., 2020). Similarly, exosome-mediated delivery of miR-144 to cardiomyocytes effectively reduces hypoxia-induced apoptosis by inhibiting PTEN and activating AKT signalling (Wen et al., 2020). Another important regulator, miR-182-5p, directly targets Gasdermin D, a central mediator of pyroptosis (Yue et al., 2022). In addition, MSC-derived exosomes provide myocardial protection in the early stages of myocardial ischemia/reperfusion injury by delivering miR-132-3p, which modulates the PTEN/AKT pathway to enhance glucose uptake and utilisation in cardiomyocytes (Wu et al., 2024).

In both *in vitro* and *in vivo* models, earlier experimental studies of ExomiRs transfer via stem cells reported several outcomes, including reduced inflammatory cell death, reduced reactive oxygen species accumulation, reduced infarct size, and improved cardiac function (Ma et al., 2018). miR-132 enhanced vascular regeneration by inhibiting RAS p21 protein activator 1, thereby stimulating angiogenesis and improving post-infarction cardiac performance (Ma et al., 2018). miR-181a has been reported to mitigate ischemia-reperfusion injury *in vivo* (Wei et al., 2019), meanwhile, miR-338 decreases apoptosis through the regulation of the Mitogen-Activated Protein Kinase Kinase Kinase 2/c-Jun N-terminal Kinase signalling pathway (Fu et al., 2020). Consequently, enhanced cardiac recovery was observed in rat models (You et al., 2024). Inhibiting apoptosis in cardiomyocytes also provided an additional protective mechanism when let-7i-5p was delivered via exosomes (You et al., 2024). On the other hand, miR-93-5p-enriched exosomes from adipose-derived stromal cells (ADSC) significantly improve cardioprotection (Liu et al., 2018). Overall, cardiomyocytes are protected by MSC- and ADSC-derived exosomal miRNAs through pleiotropic actions, which also facilitate the functional recovery of the heart. Compared with stem cell transplantation, exosome-based therapies offer enhanced safety and feasibility.

## 6 Conventional biomarkers of acute coronary syndrome

Cardiac biomarkers have significantly evolved for the diagnosis of ACS. Certain highly specific and sensitive cardiac biomarkers, like cardiac troponins, such as cTnI, cardiac troponin T, and Creatine Kinase-Myocardial Band (CK-MB), offer high-level diagnostic precision and remain central to clinical evaluation (Welsh et al., 2019; Liu et al., 2024). With further advancements, improved ACS diagnosis for early and precise detection was achieved through high-sensitivity cardiac troponin (hs-cTn) assays, such as the fifth-generation hs-cTn (Garg et al., 2017; Mueller, 2014). In addition to these established biomarkers, novel candidates such as heart-type fatty acid-binding protein-4 (H-FABP4) have been demonstrated to be sensitive early markers of rapid release and clearance (Chacko et al., 2017). Similarly, myeloperoxidase, a promising inflammatory mediator implicated in plaque rupture, was investigated for risk stratification (Chacko et al., 2017).

Beyond their diagnostic role, prognostic insights into ACS can also be derived from supplementary biomarkers (Armstrong et al., 2006). The acute-phase marker C-reactive protein (CRP) is the most validated inflammatory biomarker and has therapeutic relevance in ACS (Armstrong et al., 2006). Furthermore, cytokines such as IL-6, monocyte chemoattractant protein-1, interleukin-18, and TNF-α are strongly correlated with plaque instability and remodelling, while interleukin-10 demonstrates an anti-inflammatory effect (Armstrong et al., 2006). To enhance the early detection and risk prediction, several emerging markers, like cystatin-C and H-FABP4, and neurohormonal markers like N-terminal pro-B-type natriuretic peptide (NT-proBNP) and copeptin, were utilised (Eggers and Lindahl, 2017). Alternatively, thrombosis-related biomarkers such as D-dimer and fibrinogen indicate hypercoagulability and adverse outcomes after PCI (Eggers and Lindahl, 2017). Recently identified biomarker candidates such as endocan, galectin-3, growth-differentiation factor-15, soluble suppression of tumorigenicity 2, F2-isoprostanes, and soluble lectin-like oxidised low-density lipoprotein receptor-1 capture pathophysiological pathways and hold potential for improved risk stratification and therapeutic approaches (Eggers and Lindahl, 2017; Filippatos et al., 2014). However, most of these oxidised markers are still in the research phase, so troponins and imaging continue to dominate the standard routine for ACS diagnosis.

## 7 Limitations of conventional biomarkers and the emerging role of miRNAs in acute coronary syndrome diagnosis

Even though cardiac troponins and CK-MB remain the current gold standard due to their high sensitivity, their delayed elevation after symptom onset limits their use for early diagnosis (Chen et al., 2019). They are also elevated in various non-ischemic conditions like myocarditis, heart failure, renal dysfunction, and pulmonary embolism, which further restricts their diagnostic specificity (Omran et al., 2022). CK-MB lacks cardiac specificity and can yield false-positive results due to its expression in non-cardiac tissues (Omran et al., 2022). Similarly, aspartate aminotransferase, lactate dehydrogenase, and myoglobin rise early but nonspecifically, as they originate from a variety of tissues (Tilea et al., 2021). Natriuretic peptides like NT-proBNP appeared to be a potential prognostic indicator for heart failure, but various factors like age, renal function, and obesity confound them. Although novel biomarkers such as H-FABP4 show early elevation, they often possess limited diagnostic accuracy in some studies (Khalil, 2022). Despite the wide range of available biomarkers, no single existing biomarker adequately reflects the complexity of ACS (Xu et al., 2018). This limitation supports the value of multi-marker panels and exosome-based approaches (Xu et al., 2018). Given this, miRNAs present distinct advantages for the early diagnosis of ACS (Wang et al., 2010). Due to their rapid elevation, stability in circulation, and cardiac specificity, these markers act as potential indicators of cardiac damage (Wang et al., 2010).

## 8 Merits of using exosomal miRNAs as biomarkers of acute coronary syndrome

Exosomes are secreted by various cell types, including B cells, T cells, platelets, dendritic cells, and tumour cells. They are also found in biological fluids such as plasma, serum, urine, saliva, bile, breast milk, and cerebrospinal fluid (Preethi et al., 2022). They exert signalling effects on adjacent cells and mediate long-range intercellular communication. Because of their innate capacity to cross the blood-brain barrier and cell membranes, they are considered highly promising candidates for clinical applications (Li et al., 2023). ExomiRs have emerged as promising minimally invasive biomarkers due to their remarkable stability, which protects them from RNase degradation (Valadi et al., 2007; Mitchell et al., 2008; Boukouris and Mathivanan, 2015) and their consistent detectability in body fluids (Thind and Wilson, 2016). In plasma and serum, unique miRNA profiles that are affected by cellular contamination and coagulation are also observed (Cheng et al., 2014). Nevertheless, for clinical use, serum miRNAs are preferable because they exhibit greater stability (Cheng et al., 2014). In contrast, plasma has a lower risk of leukocyte contamination than serum (Lee et al., 2020). Therefore, these miRNAs can be effectively used not only for diagnostic but also for therapeutic purposes.

## 9 Therapeutic and translational perspectives of exosomal miRNAs

Antagomirs are a category of chemically modified anti-miR oligonucleotides that provide effective, precise and sustained suppression of endogenous pathological miRNAs in both *in vitro* and *in vivo* models (Krützfeldt et al., 2007). These oligonucleotides promote miRNA degradation via a mechanism distinct from RNA interference (RNAi) and do not rely on exonuclease activity (Krützfeldt et al., 2007). They exhibit a position-dependent single-nucleotide mismatch discrimination (Krützfeldt et al., 2007). They have shown efficacy across multiple tissues in murine models and hold potential as therapeutic agents for miRNA-targeted interventions (Krützfeldt et al., 2005). Antagomirs are also effectively employed via exosome delivery in diverse disease conditions, like bone loss (Hu et al., 2021), bone fracture healing (Xu et al., 2020) and triple-negative breast cancer (Razaviyan et al., 2024). In addition, they have demonstrated improved efficacy in certain preclinical models of atherosclerosis, myocardial infarction, and cardiac hypertrophy through targeted delivery systems (Saenz-Pipaon et al., 2023). However, not all disease-associated miRNA dysregulation results from overexpression of pathogenic miRNAs. The downregulation of protective miRNAs can also contribute to disease progression. To address this, agomirs, which are chemically modified synthetic miRNA mimics, have emerged to restore the downregulated miRNAs and modulate the gene expression (Li J. et al., 2021; Liu et al., 2021). Agomir-based therapies, including miR-495-3p (Meng and Xu, 2022) and the combined miR-21/miR-146a agomirs (Huang et al., 2016) confer significant cardio protection by attenuating oxidative stress, suppressing cardiomyocyte apoptosis and improving cardiac function. Together, antagomirs and agomirs represent complementary approaches in miRNA-based therapeutics, enabling either the suppression of deleterious miRNAs or the restoration of cardioprotective miRNAs.

In a pig model of reperfused AMI, a single intracoronary injection of encapsulated antagomir-92a effectively promotes neo-angiogenesis and prevents adverse left ventricular remodelling without local or systemic effects (Bellera et al., 2014). miR-448 acts as an indicator of myocardial risk through Sodium Voltage-Gated Channel Alpha Subunit 5 suppression, while its inhibition by antagomir-448 offers cardio-protection (Kang et al., 2020). Similarly, miR-208a regulates endoglin-mediated fibrosis in AMI, which is suppressed by antagomir-208a (Shyu et al., 2015). Antagomir-mediated inhibition of miR-140-3p targeting Zonula Occludens-1 reverses endothelial dysfunction in mouse models (Han et al., 2024). ExomiR-30a expression was regulated by agomir-30a and antagomir-30a, along with mitigating a cardiac injury post-AMI (Li Y. et al., 2024). Additionally, Heat shock protein 20 has been identified as the downstream cardiomyocyte target of miR-320; overexpression of miR-320 promoted cardiomyocyte death, whereas its inhibition via antagomir-mediated knockdown showed positive results (Ren et al., 2009). Further evidence indicates that antagomir-1 regulates endothelial barrier integrity by modulating Myosin Light Chain Kinase activity and Extracellular Signal-Regulated Kinase signalling, under hypercholesterolemic conditions (Wang et al., 2013).

In a rodent model of chronic myocardial infarction, a direct inhibition of miR-181a resulted in reduced left ventricular function, fibrosis, and hypertrophy, along with modulation of fibrosis-related and hypertrophy-derived gene expression (Vaskova et al., 2020). miR-203, targeting the Cancer Antigen 125 gene, is upregulated in ACS (Guo et al., 2026). Introduction of agomir-miR-203 significantly reduces plaque deposition and thrombosis (Guo et al., 2026). Antagomir-522-3p-mediated inhibition of miR-522-3p promotes angiogenesis by upregulating Forkhead-box protein P1 expression (Li C. et al., 2024). Similarly, antagomir-19a-3p restores angiogenesis by enhancing cardiac function impaired by exosomal miR-19a-3p, which targets HIF-1α to suppress endothelial proliferation and angiogenesis after MI (Gou et al., 2020). Administration of miR-142-3p antagomir attenuated endothelial cell apoptosis and inhibited the progression of atherosclerosis in the aorta of Apo^−/−^ (Apolipoprotein E-deficient knockout) mice (Bing et al., 2018). In summary, the integration of exosome profiling and antagomir therapeutics advances precision medicine for ACS.

## 10 Conclusion

**FIGURE 3:**
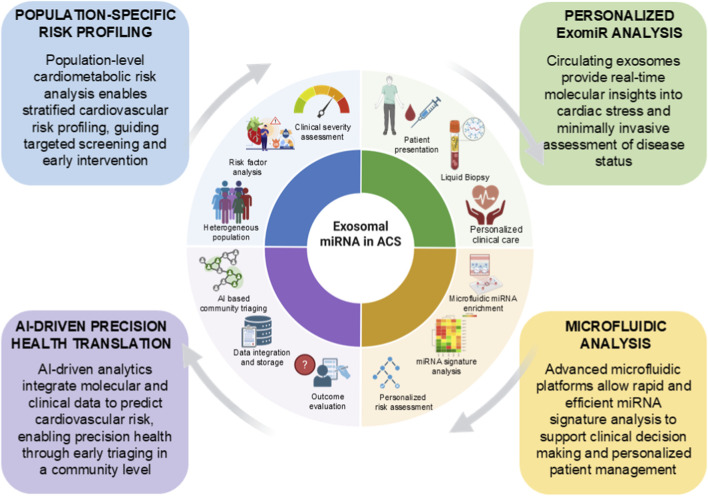
Depicts precision cardiovascular disease framework. Integration of personalized ExomiR analysis at the population-level enables improved risk assessment. Enrichment of liquid biopsy-driven ExomiRs using advanced microfluidic technologies provides real-time insights into disease severity and promotes AI-driven translation for precision healthcare.

The increasing incidence of ACS has contributed to a growing burden of cardiovascular complications with serious clinical consequences. Addressing this challenge requires not only effective clinical management but also the integration of bio-behavioural and lifestyle-based interventions to improve outcomes and reduce disease progression. Recent research has highlighted the role of ExomiRs in cardiovascular diseases, indicating their potential involvement in the pathogenesis of ACS. Unlike conventional protein-based biomarkers like cardiac troponins, which require multiple steps, including transcription and translation, before reaching detectable levels, the rapid release of ExomiRs into circulation enables early detection of molecular alterations associated with ACS (Wang et al., 2010). This approach enables effective diagnosis by supporting precision medicine and precision public health strategies. However, despite these promising attributes, the underlying molecular mechanisms remain insufficiently understood. These panels of ExomiRs can be potentially used for the early detection and risk stratification of ACS in populations characterised by diverse ethnic, socio-cultural, and lifestyle factors. This is particularly relevant in regions such as the Indian subcontinent, where heterogeneity in diet, environment, and cardiovascular risk profiles may influence biomarker expression. Identifying population-specific ExomiRs could improve early diagnosis and enable timely intervention, thereby reducing adverse outcomes (Figure 3).

At the community level, capturing and integrating cardiovascular risk signals to identify emerging ACS hotspots remains a major challenge. The application of federated learning models in conjunction with mobile health platforms offers a scalable approach for real-time monitoring and detection. Such systems can enhance early identification of high-risk individuals, support efficient allocation of healthcare resources, and provide insights into modifiable risk factors, even in underserved settings (Nandi et al., 2022; Yadav et al., 2025). AI-enabled dashboards that integrate clinical data, digital health indicators, and molecular biomarkers can further support precision-based, large-scale screening (Mitra et al., 2023). These tools facilitate remote healthcare delivery, improve patient engagement, and enable predictive surveillance, ultimately contributing to more responsive and equitable cardiovascular care systems.
